# Phosphorylation alters the mechanical stiffness of a model fragment of the dystrophin homologue utrophin

**DOI:** 10.1016/j.jbc.2022.102847

**Published:** 2022-12-29

**Authors:** Maria Paz Ramirez, Sivaraman Rajaganapathy, Anthony R. Hagerty, Cailong Hua, Gloria C. Baxter, Joseph Vavra, Wendy R. Gordon, Joseph M. Muretta, Murti V. Salapaka, James M. Ervasti

**Affiliations:** 1 Department of Biochemistry, Molecular Biology and Biophysics, University of Minnesota – Twin Cities, Minneapolis, MN, USA; 2 Department of Electrical and Computer Engineering, University of Minnesota – Twin Cities, Minneapolis, MN, USA

**Keywords:** utrophin, dystrophin, phosphorylation, atomic force microscopy (AFM), protein stability, muscular dystrophy, and mechanobiology, ABD, actin-binding domain, AFM, atomic force microscopy, CD, circular dichroism, DGC, dystrophin–glycoprotein complex, DMD, Duchenne muscular dystrophy, DMEM, Dulbecco's Modified Eagle Medium, dox, doxycycline, DysR8-15, dystrophin fragment corresponding to SLR 8 through 15, NT, amino terminal, PBST, PBS 0.1% Tween, SLR, spectrin-like repeat, UtrN-R3, utrophin fragment encoding the N terminus through spectrin repeat 3, WLC, worm-like chain

## Abstract

Duchenne muscular dystrophy is a lethal muscle wasting disease caused by the absence of the protein dystrophin. Utrophin is a dystrophin homologue currently under investigation as a protein replacement therapy for Duchenne muscular dystrophy. Dystrophin is hypothesized to function as a molecular shock absorber that mechanically stabilizes the sarcolemma. While utrophin is homologous with dystrophin from a molecular and biochemical perspective, we have recently shown that full-length utrophin expressed in eukaryotic cells is stiffer than what has been reported for dystrophin fragments expressed in bacteria. In this study, we show that differences in expression system impact the mechanical stiffness of a model utrophin fragment encoding the N terminus through spectrin repeat 3 (UtrN-R3). We also demonstrate that UtrN-R3 expressed in eukaryotic cells was phosphorylated while bacterial UtrN-R3 was not detectably phosphorylated. Using atomic force microscopy, we show that phosphorylated UtrN-R3 exhibited significantly higher unfolding forces compared to unphosphorylated UtrN-R3 without altering its actin-binding activity. Consistent with the effect of phosphorylation on mechanical stiffness, mutating the phosphorylated serine residues on insect eukaryotic protein to alanine decreased its stiffness to levels not different from unphosphorylated bacterial protein. Taken together, our data suggest that the mechanical properties of utrophin may be tuned by phosphorylation, with the potential to improve its efficacy as a protein replacement therapy for dystrophinopathies.

Duchenne muscular dystrophy (DMD) is a lethal muscle wasting disorder that affects 1 out of 4000 boys born in the US (1). DMD is characterized by muscular weakness and degeneration (2), leading to loss of motor function; boys are wheelchair-bound in their early teens and die around their late twenties (3). DMD results from the absence or reduction of the protein dystrophin, caused by an array of gene mutations, which leads to muscle cell membrane (sarcolemma) fragility, rupture, and ultimately myofiber death (2).

Dystrophin is a 427 kDa subsarcolemmal protein most abundantly expressed in skeletal muscle where it is part of a multiprotein adhesion complex called the dystrophin–glycoprotein complex (DGC) (4). The DGC is a transmembrane complex that links the intracellular actin cytoskeleton to extracellular matrix components *via* dystrophin (5), maintaining the structural stability of the cell membrane (3). Dystrophin is composed of four major domains: an amino terminal (NT) actin-binding domain (ABD1) (6, 7), a central rod domain composed of 24 spectrin-like repeats (SLRs) (8, 9) with four flexible hinges interspersed (10), which include a second actin-binding domain (ABD2) (11) among others, a cysteine-rich domain (9) that binds to β-dystroglycan (12, 13), finally followed by a carboxy-terminal domain (14) (Fig. 1*A*). Dystrophin also interacts with microtubules *via* two microtubule organizing domains (15, 16) and binds to signaling molecules such as the neuronal nitric oxide synthase (17) *via* α-syntrophin (18, 19, 20) (Fig. 1*A*).

**Figure 1:**
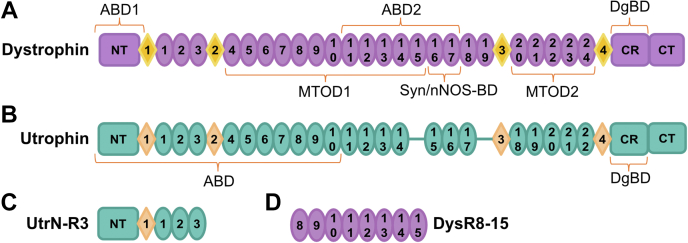
**Dystrophin and utrophin.***A,* diagram of full-length dystrophin. *B,* full-length utrophin. *C,* UtrN-R3 construct. *D,* DysR8-15 construct. ABD1 & 2, actin-binding domains; CR, cysteine-rich domain; CT, C terminus; *circles*, spectrin-like repeats, *diamonds,* disordered hinge regions; DgBD, dystroglycan binding domain; MTOD 1 & 2, microtubule organizing domain; nNOS BD, neuronal nitric oxide synthase binding domain; NT, N terminus; Syn, syntrophin; UtrN-R3, utrophin fragment encoding the N terminus through spectrin repeat 3.

Dystrophin stabilizes the sarcolemma and protects muscle against mechanical forces; however, the molecular mechanisms of this protection remain unclear. It is hypothesized that dystrophin functions as a molecular shock absorber, where SLRs reversibly unfold to dissipate energy upon muscle stretching and contraction (3, 21). Several proposed therapies to treat DMD involve expression of proteins that can substitute for dystrophin in its biochemical and biophysical functions at the sarcolemma, such as engineered miniaturized versions of dystrophin that contain a selection of critical functional domains (22) or a naturally occurring homologous protein, utrophin (23, 24). Utrophin is a fetal homologue of dystrophin, highly expressed in DMD and healthy muscle alike during fetal development, localizing to the subsarcolemma and generating a similar complex to the DGC (Fig. 1*B*) (25). After birth, dystrophin expression dramatically increases and displaces utrophin from the subsarcolemmal space, relegating it to the myotendinous and neuromuscular junctions (26). Overexpression of utrophin thus has been proposed as a potential DMD therapy and has been tested preclinically and clinically (27, 28, 29, 30); it is naturally expressed in dystrophic muscle, and its overexpression can compensate for dystrophin deficiency in mice (31, 32). Multiple studies show that utrophin is a suitable biochemical match for dystrophin in muscle (25, 33, 34), but it is unknown if it can mechanically replace dystrophin and protect the sarcolemma.

Two previous single molecule force spectroscopy studies show that model dystrophin fragments behave as soft springs with unfolding forces of ∼20 pN (21, 35), comparable to those for spectrin (36, 37). In contrast, our data revealed that full-length utrophin and large fragments encoding the N- and C-terminal halves unfolded with forces over 100 pN (38). Such forces are similar to the unfolding forces for PEVK and Ig-like domains of titin (37). Our data suggested that utrophin may be too stiff to substitute for dystrophin as a spring or shock absorber to protect the sarcolemma and may instead act as a passive elastic element to ensure mature myofibrils span the muscle fiber linearly from tendon to tendon (39). However, our mechanical studies of utrophin utilized proteins expressed in eukaryotic insect cells while previous studies of spectrin and dystrophin utilized proteins expressed in bacteria (Fig. S1), raising the question of whether differences in cell expression system, such as posttranslational modifications, may explain differences between dystrophin and utrophin in mechanical stiffness.

Here, we demonstrate that a model utrophin fragment corresponding to the NT domain through SLR 3 (UtrN-R3, Fig. 1*C*) exhibits different mechanical stiffness when expressed in bacteria *versus* eukaryotic insect cells. We also present data supporting that phosphorylation on serine 19 (S19) and 295 (S295) of insect UtrN-R3 can explain its greater mechanical stiffness compared to bacterial UtrN-R3, which is not phosphorylated. Furthermore, we studied a dystrophin fragment corresponding to SLR 8 through 15 (DysR8-15) and showed that although phosphorylated UtrN-R3 is stiffer than DysR8-15, nonphosphorylated UtrN-R3 has similar mechanical stiffness to DysR8-15. Our results suggest a potential mechanism by which the stiffness of utrophin (and possibly dystrophin) can be regulated *in vivo via* posttranslational modifications and that it may be possible to tune the mechanical properties of utrophin to better match those of dystrophin by modulating its phosphorylation status.

## Results

### Distinct UtrN-R3 mechanical stiffness is dependent on expression system

**Figure 2:**
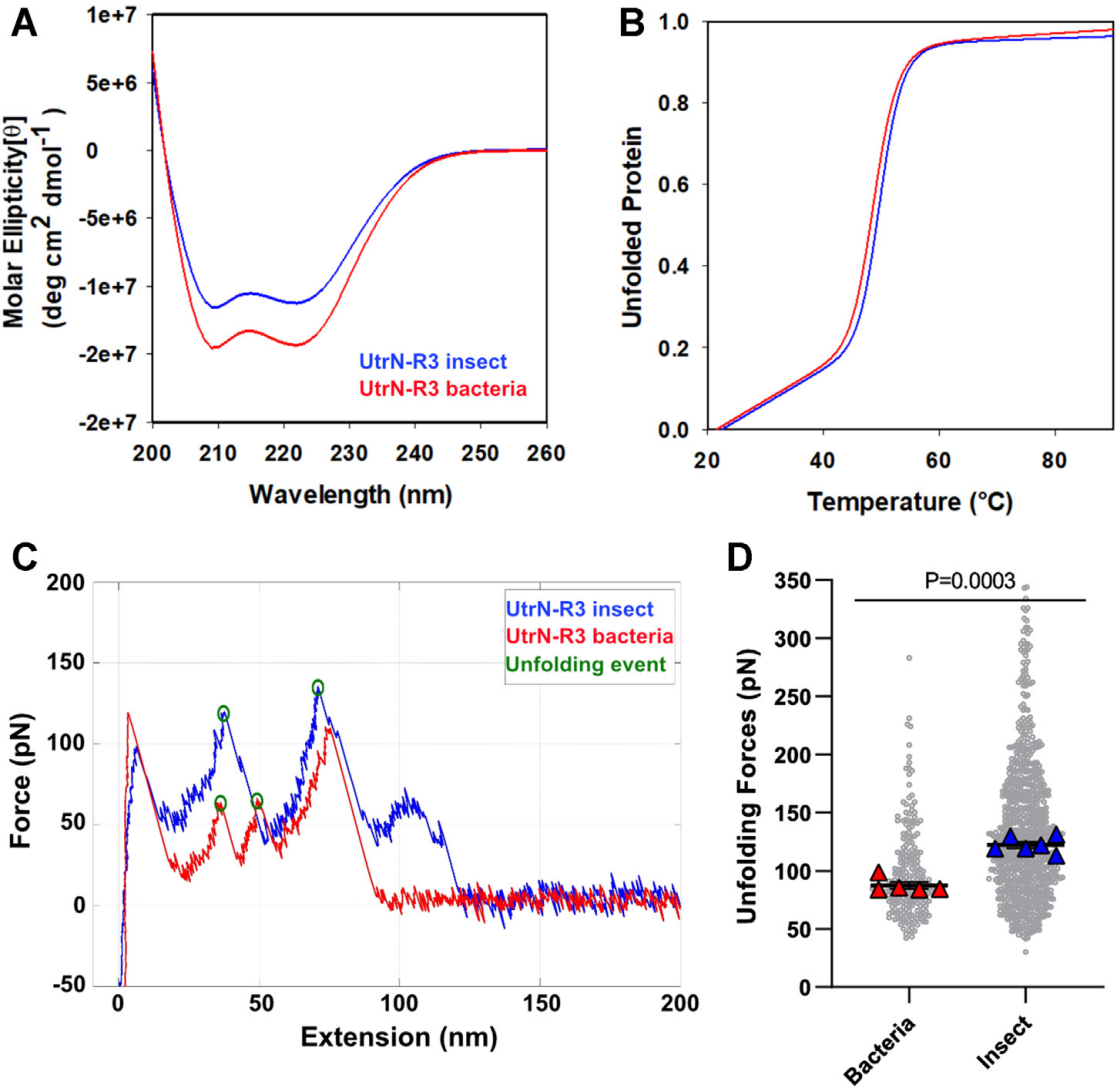
**Expression system impacts UtrN-R3 mechanical stability.***A*, CD absorption spectra from 200 to 260 nm for insect cell and bacterially derived protein. *B,* Melt curves obtained by following CD absorption spectra at 222 nm from 20 °C to 90 °C. Data are the mean of N = 3 biological replicates. Error bars represent SEM. *C,* representative force-extension curves with highlighted unfolding events of protein domains (*green circle*) *D,* measured unfolding forces for insect and bacterial UtrN-R3 from all unfolding events *(gray dots*, compilation of technical replicates). Data are median of N biological replicates (*large triangles*; N = 6). Error bars represent SEM. All data analyzed *via* unpaired 2-tailed *t* test of biological replicates; ns, not significant. CD, circular dichroism; UtrN-R3, utrophin fragment encoding the N terminus through spectrin repeat 3.

We recently demonstrated high unfolding forces for an array of utrophin fragments, including full-length utrophin (38). All utrophin constructs were expressed and purified from insect cells (Sf9 cells, derived from *Spodoptera frugiperda*), while previous mechanical studies of dystrophin were performed on protein fragments purified from bacteria (21, 35, 40). Thus, we compared the conformational stability of a utrophin construct, UtrN-R3 (Fig. 1*C*), expressed in bacteria and insect cells. To examine the effects of the expression system on UtrN-R3 *in vitro* secondary structure and thermal stability, we performed circular dichroism (CD) spectroscopy on the purified constructs. Both constructs exhibited similar spectra with strong minima at 208 and 222 nm, characteristic of structures with a high alpha-helical content (Fig. 2*A*). By following insect and bacterial UtrN-R3 spectra at 222 nm over increasing temperatures, where the constructs loss structure as they unfolded, we found that their thermal stabilities were nearly identical. Both melts curves had a single transition with melting temperatures (Tm) of 50.0 ± 0.5 °C and 48.8 ± 1.1 °C, respectively (Figs. 2*B*, 4*D*, Table 1).

**Table 1:** UtrN-R3 constructs conformational stabilities measured *via* CD and AFM

Construct	Origin	Tm (°C)	*p*-value	Unfolding forces (pN)	*p*-value
UtrN-R3 WT	Insect	50.0 ± 0.5	−	122.3 ± 2.8	−
UtrN-R3 WT	Bacteria	48.8 ± 1.1	0.9716	87.3 ± 2.9	0.0002
UtrN-R3 S19A	Insect	50.6 ± 1.2	0.9994	95.5 ± 3.4	0.0312
UtrN-R3 S168A	Insect	49.4 ± 0.5	0.9997	110.7 ± 9.4	0.7844
UtrN-R3 S295A	Insect	51.2 ± 0.4	0.9626	86.44 ± 8.9	0.0006
UtrN-R3 S295E	Bacteria	49.2 ± 0.6	0.9978	102.3 ± 3.9	0.1898
UtrN-R3 S19/295E	Bacteria	50.7 ± 0.4	0.9984	102.3 ± 3.9	0.9886
UtrN-R3 S19/168/295A	Insect	48.7 ± 0.1	0.9804	87.4 ± 3.8	0.0030

Values for Tm are mean ± SEM.

Values for unfolding forces are median ± SEM.

N = 3 for Tm and N ≥ 3 unfolding forces for each construct. Statistics were performed using one-way ANOVA, with *p* < 0.05 considered significant. All statistics were performed on biological replicates (N).

Reported *p*-values are for constructs compared against UtrN-R3 WT insect.

To study their mechanical stability, we used atomic force microscopy (AFM) to pull on single UtrN-R3 proteins at constant speed. Fig. 2*C* shows AFM force-extension curves with representative unfolding traces for each construct, where the peaks circled in green represent unfolding events of a typical folding domain. In contrast to our CD data, AFM results show that the unfolding forces for insect cell- and bacteria-derived protein were in the ranges of 122.3 ± 2.8 pN and 87.3 ± 2.9 pN, respectively (Figs. 2, *C*, *D*, and S2–S5). Our results thus demonstrate that the stiffness of UtrN-R3 is significantly impacted by the cell system employed to express it without affecting thermal stability, where insect cell–derived UtrN-R3 is stiffer than bacterial UtrN-R3.

### Eukaryotic UtrN-R3 is phosphorylated

**Figure 3:**
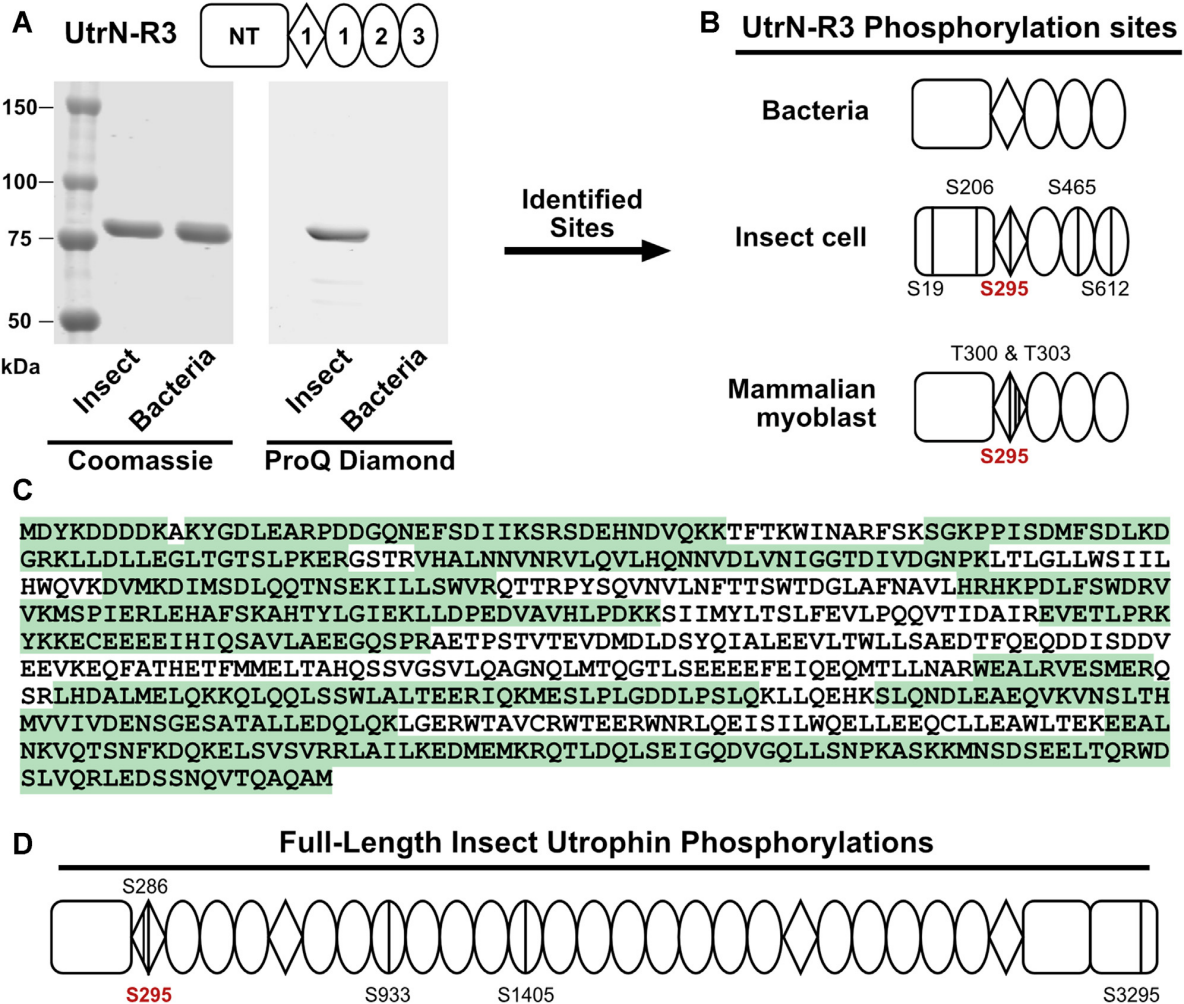
**Eukaryotic utrophin is phosphorylated.***A,* schematic of utrophin fragment analyzed *via* gels and mass spectrometry (*top*). Fragment spans the NT domain, hinge 1, and the first three spectrin-like repeats—UtrN-R3. Coomassie (*left*) and Pro-Q Diamond (*right*) stained gels of UtrN-R3 expressed in insect cells and bacteria. Total of 2 μg of total protein per lane*. B,* phosphorylation sites identified *via* mass spectrometry for UtrN-R3 purified from bacteria, insect cells, and C2C12 mammalian myoblasts, indicated by a line and residue number *C,* insect UtrN-R3 mass spectrometry coverage. *D,* phosphorylation sites identified from full-length utrophin purified from insect cells. Serine residue in *red* indicates the conserved phosphorylated residue across constructs. NT, amino terminal; UtrN-R3, utrophin fragment encoding the N terminus through spectrin repeat 3.

We hypothesized that differences in posttranslational modification machinery between eukaryotes and bacteria could potentially explain observed differences in mechanical properties. We expressed UtrN-R3 from three different sources, bacteria, insect, and mouse myoblasts cells (Figs. 3*A*, and S6) and assessed phosphorylation. We chose to focus on phosphorylation given previous reports of the N-terminal fraction of utrophin being phosphorylated in humans, though phosphorylation on SLRs, and the carboxy-terminal domain have been reported as well (41, 42, 43, 44). To first probe for phosphorylation, we employed Pro-Q Diamond phosphoprotein stain, which strongly stained insect UtrN-R3 but not bacterial UtrN-R3 (Fig. 3*A*). Mass spectrometry analysis identified several phosphorylated residues on insect and mammalian UtrN-R3 but none on bacterial UtrN-R3 (Fig. 3*B*). While mass spectrometry only covered 76% of UtrN-R3 sequence, insect cell–derived protein was phosphorylated on S19, S206, S295, S465, and S612 (Fig. 3*C*). We achieved 100% sequence coverage for mouse UtrN-R3, which identified phosphorylation on S295, T300, and T303 (Fig. 3*B*). We also analyzed full-length utrophin for phosphorylation and found phosphorylation on S286, S295, S933, S1405, and S3295 (Fig. 3*D*). Consistent with our data, a large phosphoproteomic screen of human cells identified S295 phosphorylation on human utrophin (41, 42), thus demonstrating that phosphorylation of S295 is conserved across different organisms and cell types.

### Preventing or mimicking phosphorylation at S19 and S295 alters UtrN-R3 stiffness

**Figure 4:**
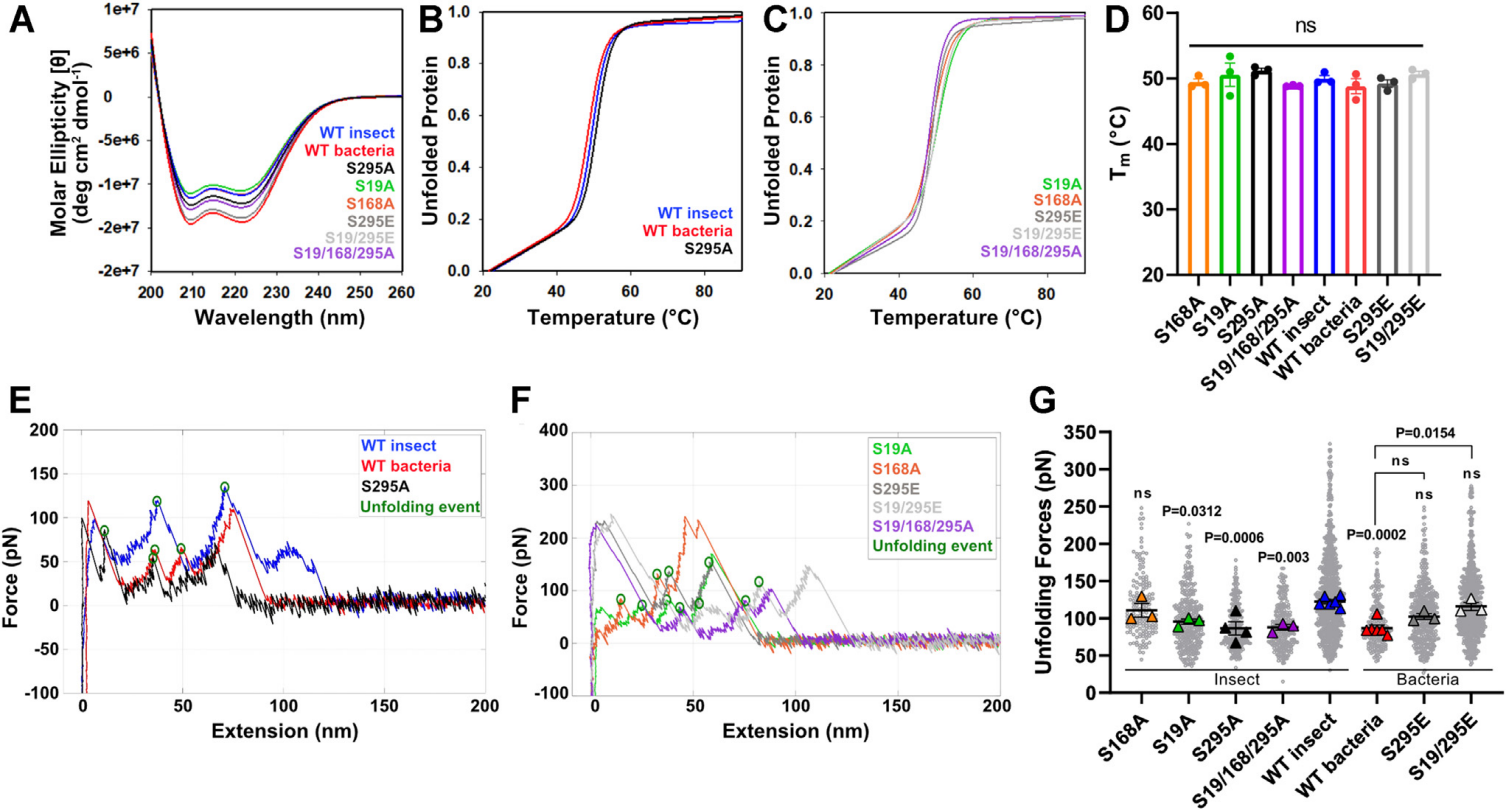
**Ablation of serine phosphorylation decreases UtrN-R3 unfolding forces.***A,* CD absorption spectra from 200 to 260 nm. *B–C,* melt curves obtained by following CD absorption spectra at 222 nm from 20 °C to 90 °C. *D*, melting temperatures (Tm) for each construct. Data are the mean of N = 3 biological replicates. Error bars represent SEM. *E–F*, representative force-extension curves with highlighted unfolding events of protein domains (*green circle*). *G,* measured unfolding forces for UtrN-R3 constructs from all unfolding events (*gray dots*, compilation of technical replicates). Data are median of N biological replicates (*large triangles*; N represented by each triangle). Error bars represent SEM. Data analyzed *via* one-way ANOVA of the biological replicates; ns, not significant. Reported *p*-values directly over data points in plot correspond to the construct compared to UtrN-R3 WT insect. *p*-values over brackets correspond to comparison of paired data. CD, circular dichroism; UtrN-R3, utrophin fragment encoding the N terminus through spectrin repeat 3.

From the five serines identified as phosphorylated in insect cell expressed UtrN-R3, we generated two single serine to alanine mutants in insect cells to assess how the loss of phosphorylation affected mechanical behavior. We chose to focus on insect S19 and S295 (Figs. S7 and S8) because they were previously identified as phosphorylated in an unbiased phosphoproteomics screen (41, 42), and they were also predicted to be phosphorylated (Fig. S9). The thermal stabilities of S19A and S295A were not different from WT insect UtrN-R3 (Fig. 4, *A*–*D* and Table 1); however, each mutant exhibited unfolding forces comparable to WT bacterial UtrN-R3 (95.5 ± 3.4 pN and 86.44 ± 8.9 pN, respectively), as shown by the representative unfolding traces for each construct and the consolidated unfolding forces for all unfolding events (Figs. 4, *E*–*G*, S2–S5, and Table 1), suggesting that phosphorylation at S19 and S295 impact the mechanical stiffness of insect UtrN-R3. As a control, we mutated serine 168 to an alanine (S168A), which was not found to be phosphorylated *via* mass spectrometry (Figs. S7 and S8). None of the biophysical parameters measured for insect S168A were significantly different from those of WT insect UtrN-R3, with Tm 49.4 ± 0.5 °C and unfolding forces in the range of 110.7 ± 9.4 pN (Figs. 4, S2–S5, and Table 1), indicating that the alterations in mechanical stiffness for insect S19A and S295A were due to the loss of phosphorylation. The effects of replacing S19 and S295 to an alanine were of similar magnitude and not statistically different.

To explore the effect of multiple phosphorylations, we made an insect cell expressed triple mutant based on the single mutants, S19/168/295A (Figs. S7 and S8) and probed its thermal and mechanical stability *via* CD and AFM. CD shows that the secondary structure content and thermal stability (Fig. 4, *B*–*D* and Table 1) were similar to WT insect cell expressed UtrN-R3 and single mutant constructs (Fig. 4*A*). AFM data show that S19/168/295A mutant exhibited unfolding forces that were at comparable levels to unfolding forces exhibited by WT bacterial UtrN-R3, S19A and S295A, hence the effect of simultaneous loss of phosphorylation by mutation on these residues is neither synergistic nor additive.

Finally, to mimic phosphorylation, we expressed and purified single (S295E) and double (S19/295E) serine to glutamic acid mutant versions of UtrN-R3 in bacteria (Figs. S7 and S8). Both constructs showed CD spectra and thermal stability that were not different from WT bacterial UtrN-R3 (Fig. 4, *A*–*D* and Table 1). S295E unfolding forces were not different from insect and bacterial WT protein, 102.3 ± 3.9 pN, although it trended toward higher forces, whereas S19/295E exhibited significantly higher unfolding forces from WT bacterial UtrN-R3, 115.9 ± 5.4 pN, that were not different from WT insect UtrN-R3 (Figs. 4, *E*, *F*, S2–S5, and Table 1). Therefore, mimicking phosphorylation at S19 and S295 is necessary to increase stiffness of bacterially expressed UtrN-R3 to the stiffness of WT UtrN-R3 expressed in insect cells.

### Phosphorylation has no impact on the actin-binding activity of UtrN-R3

**Figure 5:**
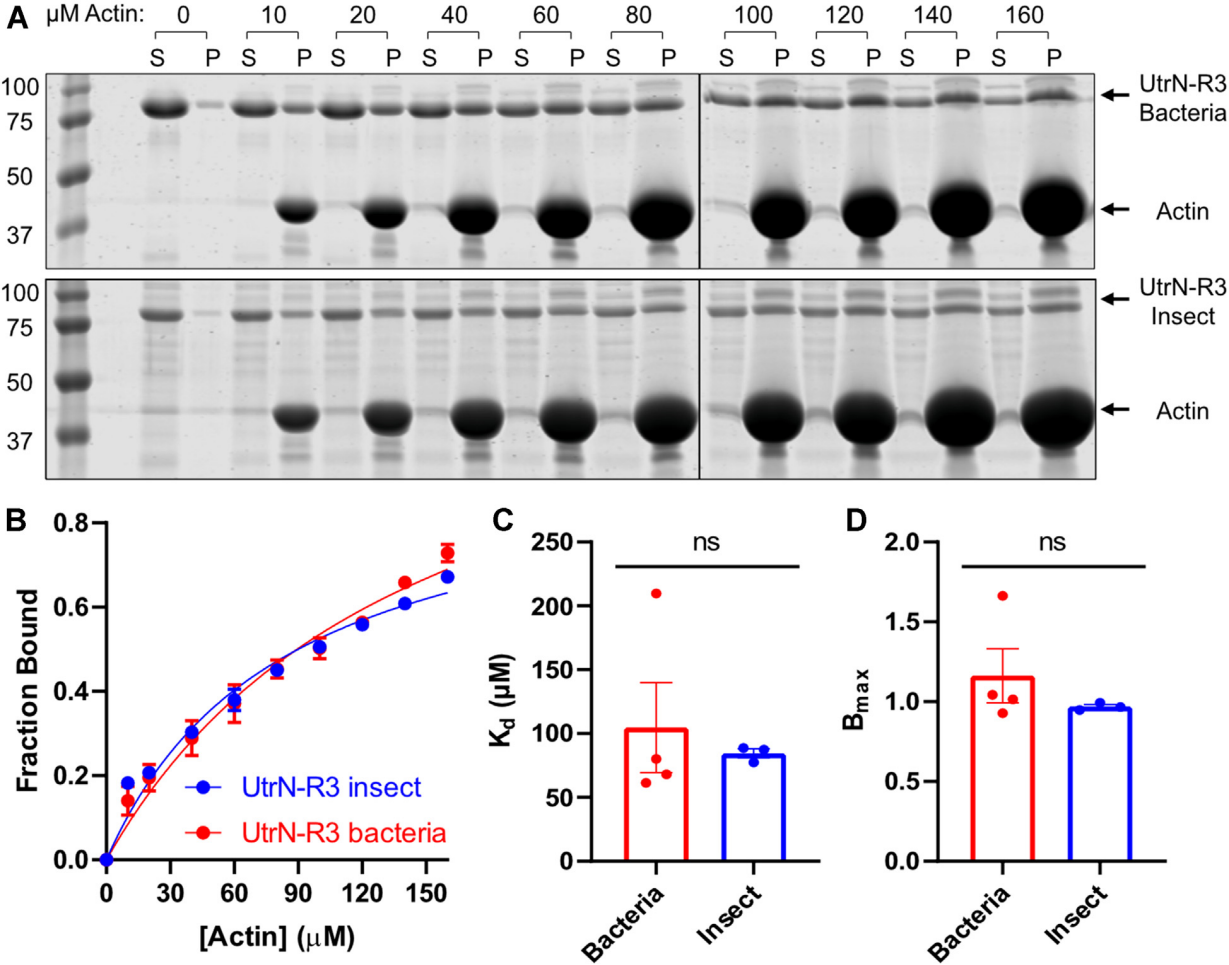
**Phosphorylation does not impact UtrN-R3 actin-binding ability.***A,* F-actin cosedimentation assay with increasing phalloidin-stabilized actin concentrations (0–160 μM) and constant concentrations of recombinant UtrN-R3 (5 μM) purified from bacteria (*upper*) and insect cells (*lower*). Molecular weight ladder (kDa) shown at the *left* of each gel. *B*, binding curves of insect (*blue*) and bacterial (*red*) UtrN-R3 with actin fitted with regression analysis (*solid line*). *C–D*, calculated binding affinities from the individual binding curves (*C*) and their B_max_ (*D*). Data analyzed *via* unpaired 2-tailed *t* test. Error bars represent SEM (N = 3 for insect and N = 4 for bacterial UtrN-R3). UtrN-R3, utrophin fragment encoding the N terminus through spectrin repeat 3.

We tested whether phosphorylation affects UtrN-R3 actin-binding activity because UtrN-R3 contains the principal ABD of utrophin (34) and S19 is located on the ABD NT flanking region, an extension before the canonical actin-binding motif which increases actin-binding affinity (45, 46). Moreover, phosphorylation on a serine residue adjacent to a homologous ABD, like S295 on hinge 1 which resides external to the actin-binding motif, alters the actin-binding affinity of L-plastin (47). We measured the actin-binding affinity of insect and bacterial UtrN-R3 using a high-speed actin cosedimentation assay (Fig. 5*A*). Insect and bacterial UtrN-R3–bound actin with K_d_‘s of 84.5 ± 3.6 μM and 104.8 ± 35.2 μM, respectively (Fig. 5, *B* and *C*), indicating that the expression system, and thus, the phosphorylation status affects mechanical behavior but not actin-binding affinity of UtrN-R3.

### The stiffness of UtrN-R3 is comparable to a model dystrophin fragment DysR8-15

**Figure 6:**
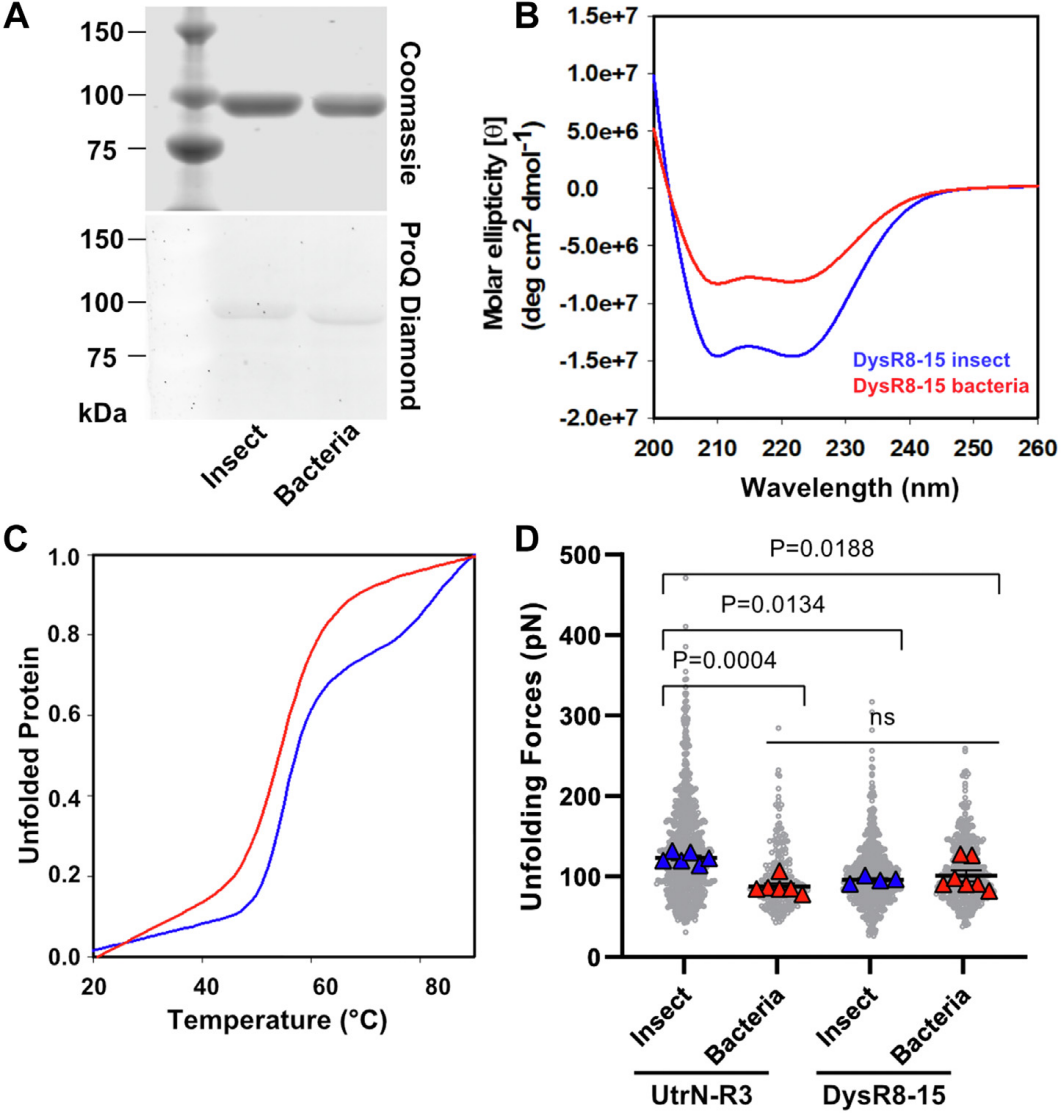
**Expression system effect on DysR8-15 mechanical stability.***A,* purified DysR8-15 fragments (97 kDa) stained with Coomassie (*top*) and Pro-Q Diamond (*bottom*). Total of 2 μg of total protein per lane. *B,* CD absorption spectra from 200 to 260 nm for insect cell (*blue*) and bacterially derived protein (*red*). *C,* melt curves obtained by following CD absorption spectra at 222 nm from 20 °C to 90 °C. Insect DysR8-15 (*blue*) presents a three-step unfolding with two intermediary states, while bacterial DysR8-15 (*red*) presents an all-or-nothing unfolding transition with 1 Tm. Absorption spectra and melt curve data are the mean of the same N = 3 biological replicates. Error bars represent SEM. *D,* measured unfolding forces for insect and bacterial constructs from all unfolding events (*gray dots*, compilation of technical replicates). Data are median of N biological replicates (*large triangles*; N represented by each *triangle*). Error bars represent SEM. All data analyzed *via* one-way ANOVA of the biological replicates; ns, not significant. CD, circular dichroism; DysR8-15, dystrophin fragment corresponding to SLR 8 through 15.

Because our data report that bacterial UtrN-R3 is still ∼5-fold stiffer than previously studied bacterially expressed dystrophin fragments (21, 35, 40), we expressed purified and characterized a dystrophin fragment corresponding to SLR 8 through 15 (DysR8-15, Fig. 1*D*) designed to match the construct studied by Bhasin *et al.* (35). Pro-Q Diamond staining and mass spectrometry data demonstrated that neither bacterial nor insect DysR8-15 was phosphorylated (Fig. 6*A*). While the CD absorption spectra were qualitatively similar for insect and bacterial DysR8-15 (Fig. 6*B*), insect and bacterial DysR8-15 protein exhibited different unfolding thermal transitions (Fig. 6*C* and Table 2) (Tm_1_ = 54.8 ± 0.1 °C and Tm_2_ = 80.4 ± 2.6 °C, T_m_, 55.2 ± 0.4 °C, respectively). However, the stiffness of insect cell and bacterial DysR8-15 was not significantly different (Figs. 6*D* and S10–S12), with median of 95.5 ± 2.1 pN and 100.1 ± 7.0 pN for insect and bacterial constructs, respectively. Our measured unfolding forces for DysR8-15 are more similar to bacterial UtrN-R3 (Fig. 6*D*) than they are to the unfolding forces reported by (35). We conclude that when compared within the same experimental system, the mechanical stiffnesses of utrophin and dystrophin are more similar than from inferences drawn from AFM data collected by different laboratories.

**Table 2:** UtrN-R3 *versus* DysR8-15 conformational stabilities measured *via* CD and AFM

Construct	Origin	Tm1 (°C)	Tm2 (°C)	*p*-value	Unfolding forces (pN)	*p*-value
UtrN-R3	Insect	50.0 ± 0.5	−	−	122.3 ± 2.8	−
UtrN-R3	Bacteria	48.8 ± 1.1	−	0.6122	87.3 ± 2.9	0.0004
DysR8-15	Insect	54.8 ± 0.1	80.4 ± 2.6	-	95.5 ± 2.1	0.0134
DysR8-15	Bacteria	55.2 ± 0.4	-	0.9653	100.1 ± 7.0	0.0188

Values for Tm are mean ± SEM.

Values for unfolding forces are median averages ± SEM.

N = 3 for Tm and N ≥ 4 unfolding forces for each construct. Statistics were performed using one-way ANOVA, with *p* < 0.05 considered significant. All statistics were performed on biological replicates (N).

Reported Tm *p*-values are bacterial construct compared against their insect counter part.

Reported unfolding forces *p*-values are the construct compared against UtrN-R3 WT insect.

## Discussion

The unfolding forces we recently reported for utrophin (38) were significantly higher than those reported for dystrophin fragments (21, 35); however, our experiments were performed on proteins expressed in eukaryotic insect cells while dystrophin fragments were expressed in bacteria. Here, we show that the cell expression system and phosphorylation impact the mechanical stiffness of a model utrophin construct. Our AFM data demonstrate that recombinant insect cell–expressed UtrN-R3 exhibits increased mechanical stiffness compared to recombinant bacterially expressed UtrN-R3 (Fig. 2). Mass spectrometry analysis demonstrate that UtrN-R3 is phosphorylated when expressed in insect and mouse cells, but not in bacteria (Fig. 3), while other proteomic studies have reported phosphorylation of utrophin expressed in human tissues and cells (41, 42). While neither bacterial nor insect DysR8-15 were detectably phosphorylated and exhibited similar unfolding forces (Fig. 6*D*), both were more comparable to UtrN-R3 in mechanical stiffness than to data reported previously (21). Our results underscore the importance of directly comparing mechanical characteristics of different proteins within the same experimental system and suggest that dystrophin and utrophin are more alike in terms of mechanical stiffnesses than hypothesized from studies performed across different laboratories.

While the exact mechanism by which phosphorylation alters the mechanical stiffness of UtrN-R3 remains to be elucidated, phosphorylation is known to alter the stability of proteins *via* phosphate-mediated electrostatic interactions with other residues. Phosphorylation can increase electrostatic repulsion/attraction or induce reorganization or *de novo* formation of salt-bridges. For example, it has been shown that formation of salt-bridges between phosphoserine (i position) and positively charged side chains in an i + 4 position can stabilize an alpha-helix (48). Insect UtrN-R3 phosphoserine 19 is located in an alpha-helix within utrophin’s NT ABD. A lysine (K23) residue can be found at position i + 4, which argues for a formation of a salt-bridge between S19 and K23 *via* a phosphate on S19. Interestingly, the conserved phosphorylation on S295 is located in hinge 1 (Fig. 3), which is a proposed unstructured region of the protein (10). Previous work has shown that intrinsically disordered protein regions affect the stability and functionality of ordered regions of the proteins (49), and phosphorylation has been shown to modulate their functionality and stability (50, 51). Intriguingly, intrinsically disordered protein regions can be conditionally disordered, with phosphorylation as a regulatory switch between order and disorder (52, 53). We speculate that such a mechanism could explain how phosphorylation of S295 in hinge 1 may alter the mechanical stiffness of UtrN-R3.

While both S19 and S295 lie outside of the tandem calponin homology domains responsible for direct binding of utrophin/UtrN-R3 to actin filaments, previous studies supported the possibility that phosphorylation at either or both residues could alter the actin-binding activity of UtrN-R3. First, a 28 residue N-terminal peptide preceding the tandem calponin homology domains has been shown to increase actin-binding affinity through a direct interaction between the peptide and actin (45, 46). Therefore, we hypothesized that covalent attachment of phosphate to S19 could inhibit actin binding through electrostatic repulsion between phospho-S19 and acidic actin filament surface. As precedent for a potential effect of phosphorylation at S295 in hinge 1 of UtrN-R3 on actin-binding activity, the actin-binding affinity of L-plastin is increased by phosphorylation of a serine located in sequence adjacent to its tandem calponin homology ABD (47). However, we found that bacterial and insect UtrN-R3–bound F-actin with K_d_ and B_max_ values were not significantly different (Fig. 5), indicating that phosphorylation does not alter actin-binding activity.

In summary, the principal finding of our study is that phosphorylation can alter the mechanical stiffness of a model utrophin fragment without impacting other aspects, such as thermal conformational stability and actin-binding function. Interestingly, the flexible PEVK domains of titin when hyperphosphorylated leads to protein stiffening, contributing to heart failure due to myofilament stiffening (54). Ablation of serine 52 phosphorylation on keratin 18 by mutation to an alanine residue leads to protein softening in cancer cells (55). We speculate that phosphorylation may provide a mechanism to tune the mechanical properties of utrophin, possibly to improve its functionality as a replacement therapy for dystrophin in DMD.

## Experimental procedures

### Cloning

Mouse utrophin N-R3 (UtrN-R3) and mouse dystrophin R8-15 (DysR8-15) truncation constructs were cloned from an existing full-length mouse utrophin and dystrophin vectors with an N-terminal FLAG-tag for use in purification (56), as described in (38). Mutant UtrN-R3 constructs were cloned *via* Gibson assembly using NEBuilder HiFi DNA Assembly (New England Biolabs), using a gblock(s) insert containing the specified mutation(s) and a backbone PCR amplified from the wildtype construct. p2Lox-mUTR-N-R3 construct, with UtrN-R3 flanked by two lox sites, was also cloned *via* Gibson assembly, with the insert and backbone PCR amplified from expression constructs containing mUTRN-R3 and the p2lox recombination sites (57). PCR primers were designed according to Gibson assembly requirements for homology-based reaction.

### Baculovirus stock

High-titer viral stocks were generated through successive infections of Sf9 cells in 3.5-cm plates (P0), 10-cm plates (P1), and 250 ml of 1 × 10^6^ cells/ml suspended cells (P2) at 28 °C. Briefly, 2 × 10^6^ total Sf9 cells in Sf-900 II SFM (Thermo Fischer Scientific) were plated for 30 min to allow for adhesion and subsequently transfected with bacmids purified from DH10Bac-competent *Escherichia coli* transformed with pDEST8 destination (according to the manufacturer’s protocol) using Cellfectin II (Thermo Fischer Scientific) and cultured for 4 days. Supernatant (P0) was collected and used to transfect 3 × 10^6^ adhered Sf9 cells in in Sf-900 II SFM up to 10 ml for 72 h. Supernatant (P1) was collected and used to transfect a 250 ml 1 × 10^6^ Sf9 cells/ml culture in suspension for 72 h. Supernatant (P2) was separated from cells by centrifugation at 4000*g* for 10 min, and collected supernatant was stored in light blocking tubes at 4 °C.

### iC2C12 UtrN-R3 cell line generation and culture

iC2C12 cells containing an inducible cassette exchange flanked by lox sites (Fig. S6), kindly provided by Dr Kyba (57), were cultured in Gibco Dulbecco's Modified Eagle Medium (DMEM, Thermo Fischer Scientific) supplemented with 10% fetal bovine serum (ATCC), 1% penicillin/streptomycin mixture (HyClone), and 0.1% fungizone (Thermo Fischer Scientific). After cells reached 40% confluency, cell media were replaced to supplemented DMEM with 500 ng/ml doxycycline (dox) (VWR) to induce endogenous Cre under a dox regulated locus, and cells were cultured for 24 h. Dox media were replaced with fresh media without dox 2 h before transfecting with Lipofectamine 3000 the p2lox-mUtrN-R3 plasmid. Selection of cells that underwent Cre-mediated recombination were selected with increasing amounts (0.5–2 mg/ml) of G418 (Thermo Fischer Scientific). After selection, cells were expanded in G418 1 mg/ml selective media and tested for UtrN-R3 expression by inducing with 5 μg/ml dox and following expression *via* Western blot (Fig. S6). Generated iC2C12 UtrN-R3 isogenic cells were then cultured and grown at 37 °C, 5% CO2 in supplemented DMEM.

### Protein expression and purification

For insect cell–derived protein, 250 ml Sf9 insect cells in suspension, maintained at 1 × 10^6^ cells/ml in Sf-900 II SFM, were transfected with 10 ml baculovirus containing conditioned media and cultured for 72 h postinfection to maximize protein expression. For bacterially derived protein, BL21 *E. coli* (utrophin) or Rosetta *E. coli* (dystrophin) harboring the expression plasmid were grown in selective media, and protein overexpression was induced when culture reached an *A*_600_ of 0.4 to 0.6 by incubation with 1 mM isopropyl-β-d−1-thiogalactopyranoside (Roche) for 4 h. For myoblast derived protein, 1 × 10^6^ iC2C12 cells were cultured on multiple 15 cm plates for 18 h in supplemented DMEM with 500 ng/ml dox to induce protein expression (57). Myoblasts were then collected by trypsinization.

Cells were harvested by centrifugation (insect and bacteria at 4000*g* and myoblasts at 1000*g*) for 10 min, and insect cells pellets were resuspended in lysis buffer [phosphate-buffered saline (PBS, Hyclone) containing, 100 nM aprotinin, 10 mg/ml E-64, 10 μM leupeptin, 1 mM PMSF, 1 μg/ml pepstatin, and 1 mM benzamide protease inhibitors] plus 1% Triton X-100for 30 min rotating at 4 °C. Bacterial pellet was resuspended in lysis buffer and then lysed by sonication with three bursts of 1 min using an ultrasonic homogenizer (BioLogics) set at 40% power 50% pulser. Both lysates were cleared by centrifuging 20,000*g* for 10 min at 4 °C. Both insect and bacterial cleared supernatant were applied to an anti-FLAG M2 agarose column (Sigma Aldrich), packed according to manufacturer. The loaded column was washed with 15 column volumes of molecular-grade PBS, and bound protein was eluted with elution buffer (PBS with protease inhibitors and 100 μg/ml FLAG peptide). Purified protein was dialyzed overnight in 2 L of PBS at pH 7.5 with 0.5 mM dithiothreitol (DTT, Millipore Sigma) and then concentrated using a centrifugal concentrator filter molecular weight cut-off 10 kDa (Amicon, Millipore Sigma). Protein concentration was determined *via* nanodrop using absorbance at 280 nm, and theoretical extinction coefficients calculated from the amino acid compositions for each construct. Purified products were verified on a 3 to 12% sodium dodecyl sulfate (SDS) polyacrylamide gradient gel. Gels were stained with Coomassie blue stain and samples visualized using Licor’s Odyssey Infrared Imaging System.

### Pro-Q Diamond phosphoprotein gel staining

Purified proteins (2 μg total) were run on a 3 to 12% SDS polyacrylamide gel and stained with Pro-Q Diamond as recommended by manufacturer. Briefly, gels were fixed overnight (50% methanol 10% acetic acid), stained with Pro-Q Diamond stain for 90 min and washed with destain solution (20% acetonitrile, 50 mM sodium acetate pH 4.0). Gels were visualized using the Typhoon FLA 9500 (GE Healthcare) and afterward stained with Coomassie blue stain and visualized using the LI-COR Odyssey Infrared Imaging System.

### Mass spectrometry

Purified concentrated proteins were run on a 3 to 12% SDS polyacrylamide gel and stained with Coomassie blue stain. The stained band corresponding to the purified protein was cut from the gel, stored in a tube filled with ultrapure water, and shipped to the Taplin Mass Spectrometry Facility for analysis of posttranslational modifications *via* reverse-phase liquid chromatography/tandem mass spectrometry. Samples were initially digested using trypsin, but to increase coverage of some samples, chymotrypsin was also employed.

### Experimental design and statistical rationale

For the posttranslational modification identifications determined by mass spectrometry, we submitted an N = 2 for insect UtrN-R3 and an N = 1 for bacterial UtrN-R3 as well as insect and bacterial DysR8-15. Constructs submitted for mass spectrometry analysis were assessed *via* Pro-Q Diamond. Samples that were not found to be phosphorylated *via* Pro-Q Diamond were sent for analysis only once. Data analysis and interpretation to determine phosphorylation sites was done according to Beausoleil *et al.* (58), using a probability-based scoring system (ModScore). Two scores are given to the modified residues and scores range from 1 to 1000. Scores for the same site above 19 signify that the location is confidently assigned; scores listed as 1000 signify that the assignment is considered unequivocal. Data analysis was done by the Taplin Mass Spectrometry Facility.

### Western blot analysis

Harvested cells were lysed with lysis buffer with added protease inhibitors. Lysates were centrifuged at 20,000*g* for 10 min at 4 °C, and the cleared lysates were run on 3 to 12% SDS polyacrylamide gels at 100 V for 30 min and 150 V for 1 h and transferred to a PVDF membrane at 0.8 amps for 1 h. Membranes were blocked for 1 h in 5% milk in PBS 0.1% Tween (PBST) and then incubated with primary antibody overnight at 4 °C. Primary FLAG (1:1000, Sigma Aldrich; F1804) antibody was diluted in milk PBST. Membranes were washed with PBST and then incubated with anti-mouse secondary antibody IgG Dylight 800 (1:10,000, Cell Signaling) diluted in PBST milk. Secondary antibody signal was visualized on LI-COR’s Odyssey CLx Imaging System, and band density was calculated with Image Studio Software.

### Circular dichroism

Purified protein constructs were centrifuged at 14,000*g* for 10 min at 4 °C, and the soluble fraction was diluted to 0.4 mg/with PBS. Absorption spectra were acquired at 20 °C, controlled by a Peltier device, from 200 to 260 nm. For thermal melt curves, spectra were acquired from 20 °C to 90 °C at 1 °C temperature intervals, and the melt curves were obtained by following the characteristic alpha-helical ellipticity at 222 nm. All measurements were acquired with a J-815 spectropolarimeter (Jasco). Molar ellipticity, [θ], was calculated as following

$$\left[\theta \right]=\frac{\theta }{10*c*l}$$

Where *θ* is the measured absorption, *c* is the sample molar concentration, and *l* is the path length in cm. For CD spectra, molar ellipticity (deg∗cm^2^∗dec^−1^) was plotted against wavelength for the CD spectra. For melt curve, normalized ellipticity at 222 nm was plotted against temperature, and fit by regression analysis using equations for two-state or three-state unfolding in Sigma Plot (Systat Software) (59).

### Atomic force microscopy

Single-molecule force spectroscopy experiments were performed utilizing an Oxford Instruments MFP-3D atomic force microscope. The AFM setup included a flexible cantilever with a sharp ‘V’ shaped tip, a laser-photodiode–based sensor, and a piezoelectric nanopositioner (60). We used the Olympus BL-RC-150VB cantilevers with Cr/Au coating on the tip, reflex, and probe side of the cantilever with a typical spring constant of 6 pN/nm and a tip radius of 25 ± 12 nm. Before each experiment, the spring constant was estimated by analyzing the thermal response of cantilever deflection (61). A droplet (∼100 μl) of purified protein in PBS at a dilution of 12.5 to 100 nM was incubated on a freshly cleaved mica substrate for 10 min prior to the experiment to allow for protein adsorption to the mica surface. The droplet of the protein solution was then removed, and the mica substrate washed with 100 μl of PBS to remove unadsorbed proteins. A fresh droplet of 100 μl of PBS was then added to provide the environment for the force spectroscopy experiments. We performed repeated approach-retraction cycles, at room temperature, where the tip of the cantilever was pressed against the substrate for 3 s with an indentation force of 600 to 800 pN, and then retracted at 1 μms^−1^. During such a cycle, if protein attachment was detected between the cantilever tip and mica substrate, the intermediate segment was stretched during the retraction phase of the cantilever. Since the pulling force on the molecule is balanced by the force experienced by the cantilever, the force on the protein was calculated as:

F=kd,

where *k* is the spring constant of the cantilever and d is the deflection of the cantilever. Upon forced extension of the protein, the folded protein domains unfolded stochastically, resulting in a saw-tooth pattern in the cantilever deflection *versus* separation curve (36). Protein concentration optimization for the experiments were done as previously described in Rajaganapathy *et al.* (38). Data from 160 to 400 successful force spectroscopy experiments (with at least one identifiable unfolding event) were collected for each protein construct and used to determine the statistical behavior of the unfolding forces.

### AFM data analysis

The raw data from the force spectroscopy experiments primarily consist of (1) the deflection of the cantilever and (2) distance between the base of the cantilever and the substrate. It includes data from cases when no protein was adsorbed between the tip and the substrate, as well as cases when a protein was successfully adsorbed and stretched. We use an algorithm developed in MATLAB (MathWorks) to identify successful experiments and to extract useful information from data. The algorithm determines the force applied by multiplying the deflection with the spring constant. The extension of the protein is computed, from the distance between the tip of the cantilever and the substrate. The algorithm detects characteristic peaks in the force signal larger than 3σ, where σ is the standard deviation of the noise in the force signal measured from the approach curve. The first significant peak is a result of the adhesive force between the cantilever tip and the substrate and a potential unfolding event from a protein. This is therefore discarded from data for further analysis. Similarly, the last significant peak is corrupted by a detachment event and is discarded. The remaining significant peaks are considered as unfolding events of interest. The number of significant peaks is then used to distinguish between the successful and unsuccessful experiments.

To estimate the contour lengths and the persistence lengths of the protein under study, we fit the measured force *versus* extension characteristics of the protein to a worm-like chain (WLC) model. The WLC model (36) is given by:

$$F\left(x\right)=\frac{k_{B}T}{P}\left[\frac{1}{4{\left(1-\frac{x}{L}\right)}^{2}}-\frac{1}{4}+\frac{x}{L}\right]\text{,}$$

where, F is the force applied on the protein, x is the extension, $k_{B}$ is the Boltzmann constant, T is the temperature, L is the contour length of the protein, and P is the persistence length. The model fit produces an estimate of L and P. The force-extension curves with the WLC fits generated by the MATLAB-based algorithm are then inspected manually. Force-extension traces that show abnormalities (such as more than the expected number of unfolding events, misshapen unfolding events with characteristics dissimilar to that of a WLC model) were not included for further analysis.

### Kolmogorov–Smirnov test analysis

To quantify the difference between two statistical distributions of parameters, we used the Kolmogorov–Smirnov test. The Kolmogorov–Smirnov test metric $D_{KS}$(62) between two empirical cumulative distributions, $\Phi_{a}\left(x\right)$ and $\Phi_{b}\left(x\right)$ is given by:

$$D_{KS}≔\max_{-\infty <x<\infty }\left|\Phi_{a}\left(x\right)-\Phi_{b}\left(x\right)\right|\text{.}$$

Here, the empirical cumulative distribution $\Phi_{a}\left(x\right)$ is computed from the samples $X_{1}^{n_{a}}:=\left\{x_{1},x_{2},\ldots {x_{n}}_{a}\right\}$ as:

$$\Phi_{a}\left(x\right)≔\frac{Number\;of\;elements\;in\;X_{1}^{n_{a}}\leq x}{n_{a}}$$

The metric $D_{KS}$ ranges from 0 implying maximum similarity (identical distributions) to 1 implying maximum dissimilarity.

### Actin high-speed sedimentation analysis

The actin-binding properties of UtrN-R3 recombinant constructs were measured using a high-speed co-sedimentation assay, as previously described in (11). Briefly, 5 μM of UtrN-R3 were incubated for 30 min with increasing amounts of rabbit skeletal muscle F-actin (0–160 μM) prepared as described in (63), stabilized with 1.1 M excess of phalloidin (Cayman Chemicals) and then centrifuged at 100,000*g* for 20 min (Optima MAX-XP Ultracentrifuge, Beckman Coulter). Reactions were incubated in F-buffer (Final concentration 4.5 mM Tris-HCl pH 8.0, 0.18 mM CaCl_2_, 50 mM KCl, 2 mM MgCl_2_, 1.2 mM ATP, and 0.5 mM DTT). Soluble and pellet fractions were run on a 3 to 12% SDS gel and stained with Coomassie Blue. The bound and free utrophin fractions were determined densitometrically from the stained gels using the LI-COR Odyssey Infrared Imaging System from the resulting F-actin pellet and supernatant fractions, respectively. Binding data were fitted to a hyperbola (x is concentration) by nonlinear regression analysis using the GraphPad Prism 8 software.

### Statistical analysis

All statistical calculations in main manuscript were performed using the GraphPad Prism 8 software. Statistical calculations and data analysis of supporting information were performed using MATLAB. Unfolding force data are presented as median ± standard error, and all the rest of the data are presented as mean ± standard error. Plots showing layered data were created as superplots (64). An unpaired two-tailed *t* test was performed to compare the means of variables between insect and bacterial WT data (same construct), performed at α = 0.05. An unpaired one-way ANOVA was performed to compare three or more populations, with α = 0.05 to determine significance. Significance was confirmed with a Tukey’s posthoc test, performed at α = 0.05. All AFM, CD, and actin-binding experiments were performed independently (biological replicate) at least three times, with each experiment performed on protein purified on separate dates from different induced cell pellets.

## Data availability

All data presented in this article are found either in the main text or supporting information.

## Supporting information

This article contains supporting information.

## Conflict of interest

The authors declare that they have no conflicts of interest with the contents of this article.

## Supporting information

Supporting information

Phospho_MS_UtrN-R3_insect
