# Supplementary material for: Phosphorylation alters the mechanical stiffness of a model fragment of the dystrophin homologue utrophin
Source: J Biol Chem. 2022 Dec 29;299(2):102847. doi: 10.1016/j.jbc.2022.102847 (PMC9922815; doi:10.1016/j.jbc.2022.102847)
Supplement: Supporting information [file mmc1.pdf]

Phosphorylation alters the mechanical stiffness of a model fragment of the dystrophin homologue utrophin

Maria Paz Ramirez[1], Sivaraman Rajaganapathy[2], Anthony R. Hagerty[1], Cailong Hua[2], Gloria C. Baxter[1], Joseph Vavra[1], Wendy R. Gordon[1], Joseph M. Muretta[1], Murti V. Salapaka[2], James M. Ervasti[1].

[1]Department of Biochemistry, Molecular Biology and Biophysics, University of Minnesota – Twin Cities, Minneapolis, Minnesota

[2]Department of Electrical and Computer Engineering, University of Minnesota – Twin Cities, Minneapolis, Minnesota

Materials include:

Figures S1-12

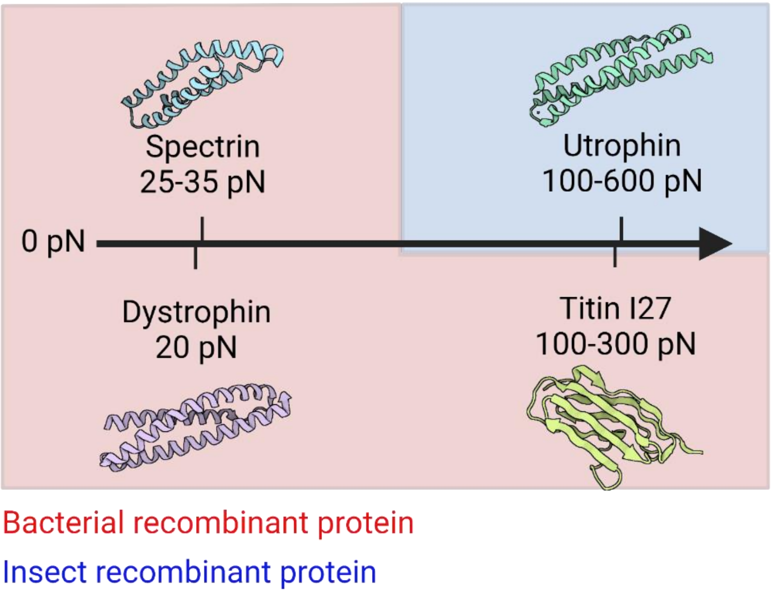

Figure S1. Mechanical Stability of Relevant Protein Relative to Dystrophin. Comparison of reported unfolding forces for dystrophin, spectrin, utrophin and titin.

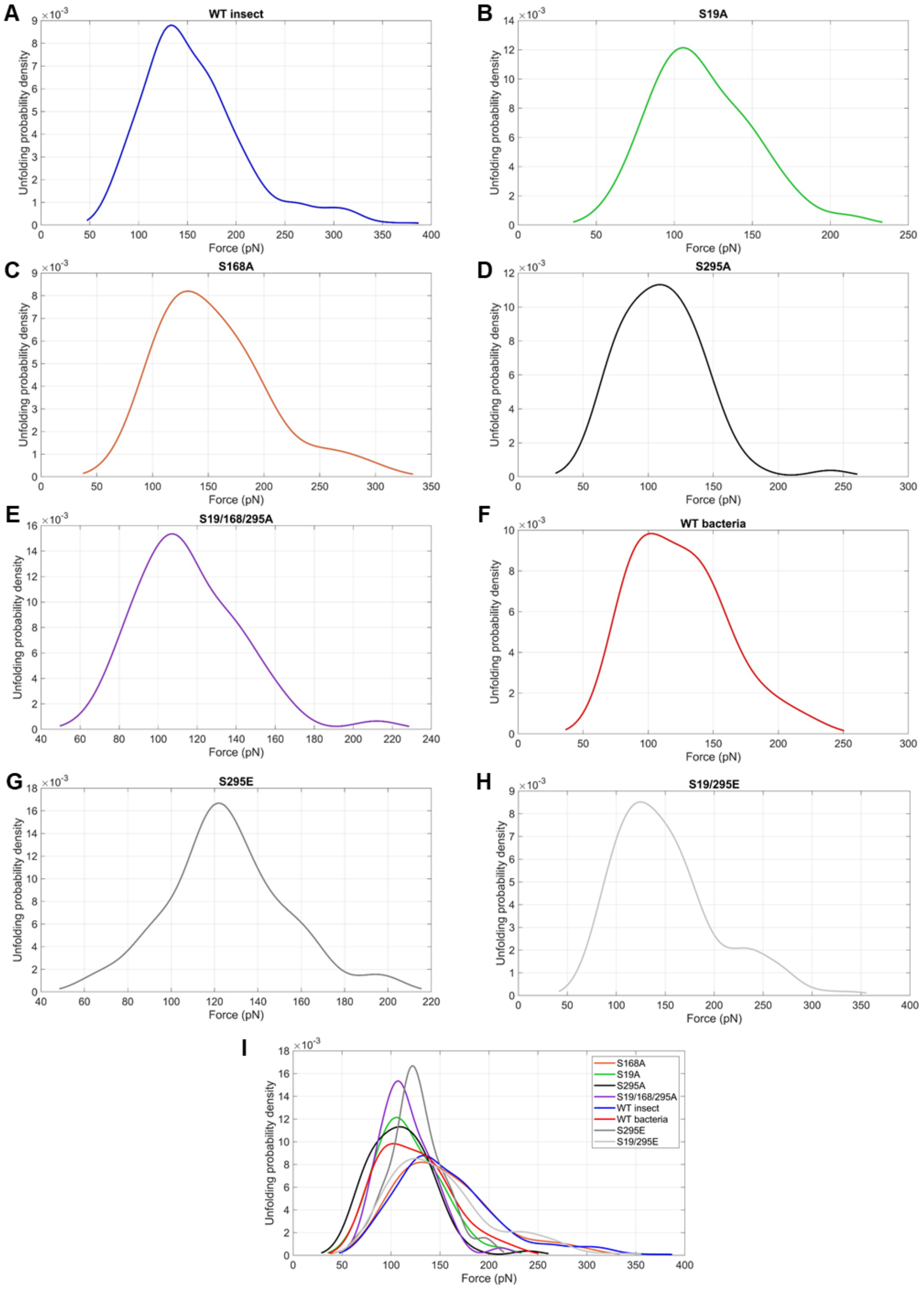

Figure S2. Unfolding force distributions for UtrN-R3. Probability density distributions of unfolding forces vs unfolding force for all UtrN-R3 constructs **A.** WT insect **B.** S19A insect **C.** S168A insect **D.** S295A insect **E.** S19/168/295A insect **F.** WT bacteria **G.** S295E bacteria **H**. S19/295E bacteria **I.** Comparative distribution between all constructs. Data collected from total unfolding events (>160 per construct), from N≥3 biological replicates.

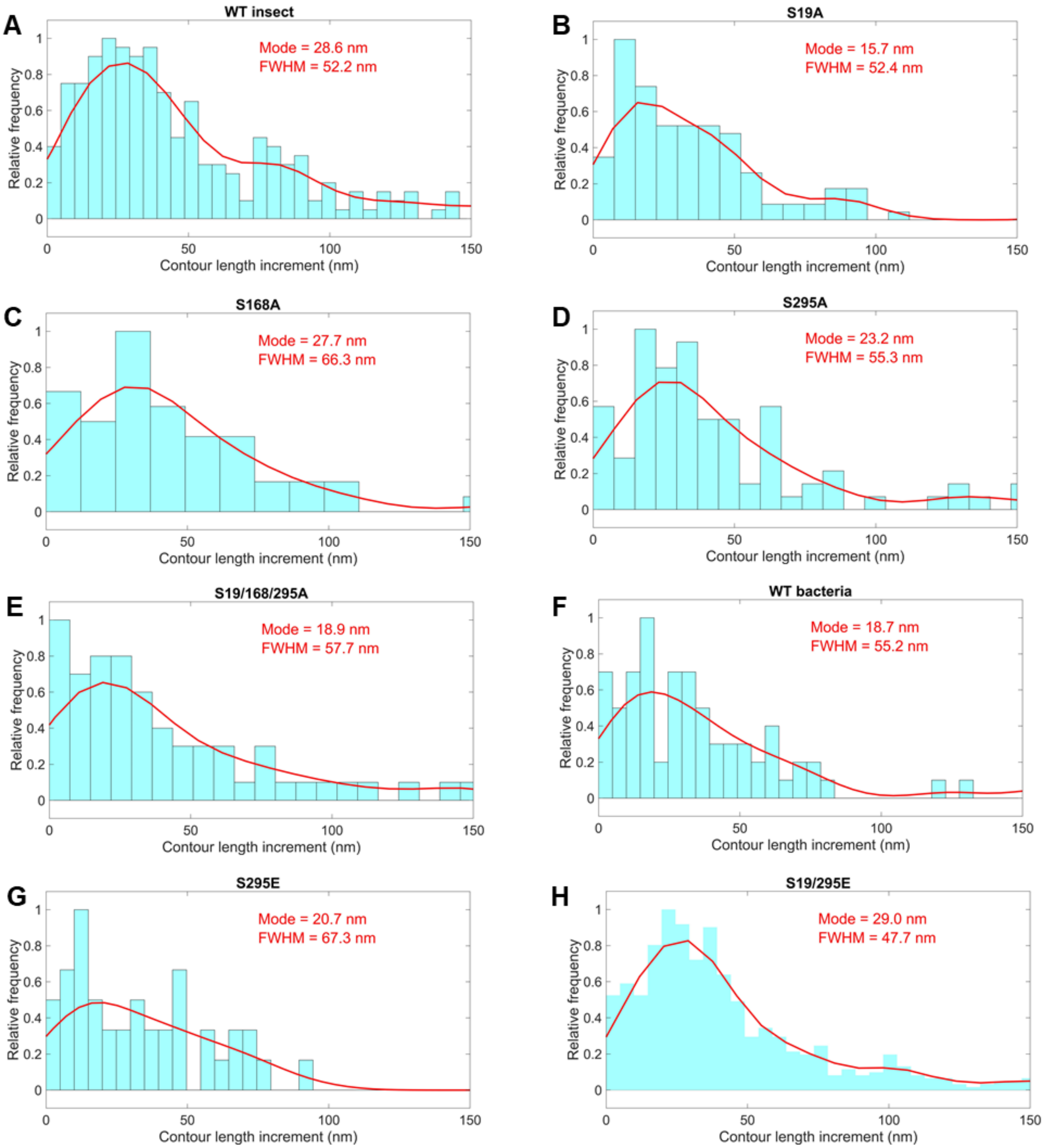

Figure S3. Contour length increment histograms for UtrN-R3 constructs. A. WT insect **B.** S19A insect **C.** S168A insect **D.** S295A insect **E.** S19/168/295A insect **F.** WT bacteria **G.** S295E bacteria **H.** S19/295E bacteria. The most probable contour length increments range between 15.7 to 29.0 nm. FWHM is full width at half maximum.

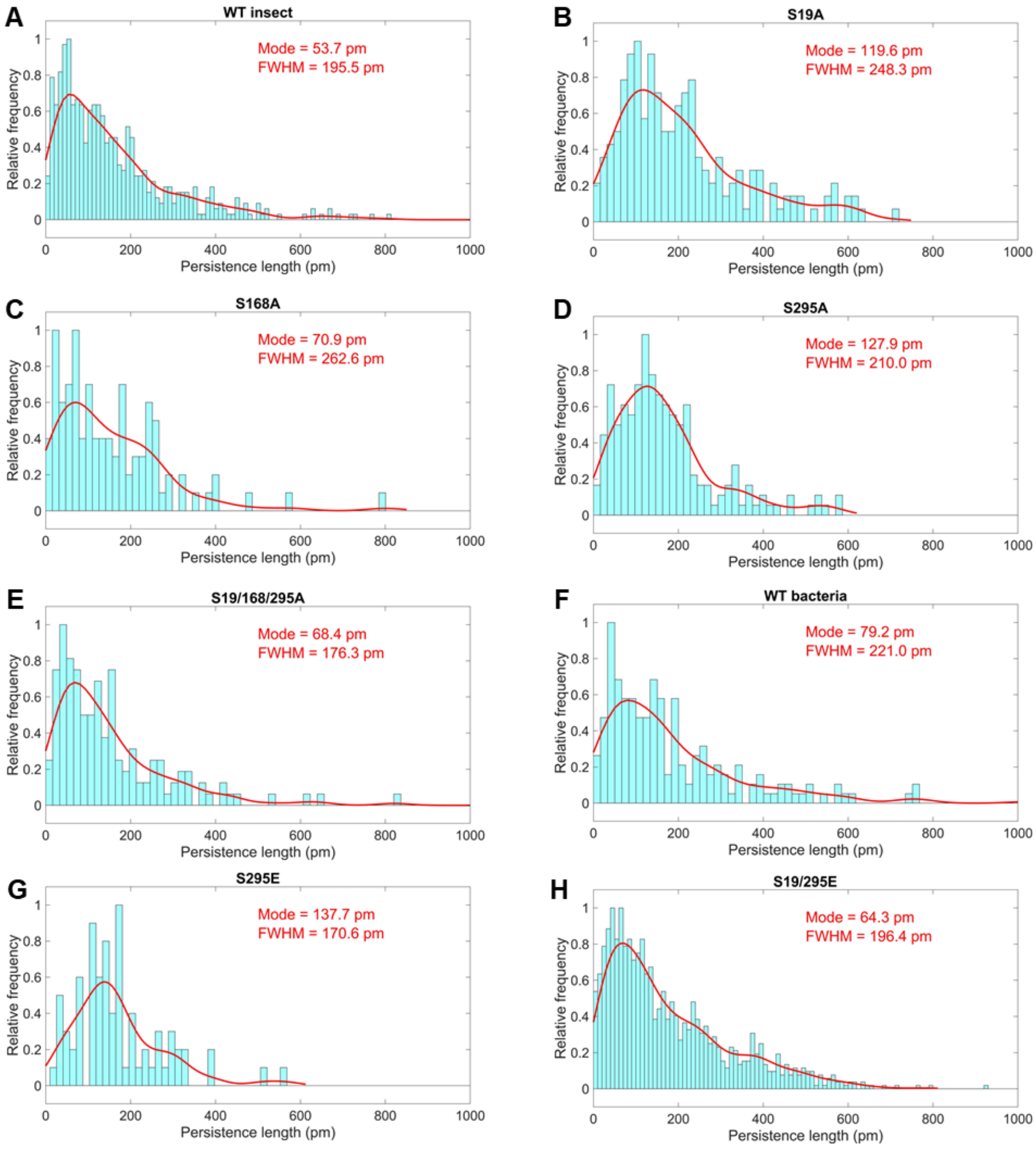

Figure S4. Persistence length increment histograms for UtrN-R3 constructs. A. WT insect **B.** S19A insect **C.** S168A insect **D.** S295A insect **E.** S19/168/295A insect **F.** WT bacteria **G.** S295E bacteria **H.** S19/295E bacteria. The most probable persistence lengths were found to be between 53.7 to 137.7 pm for the utrophin constructs.

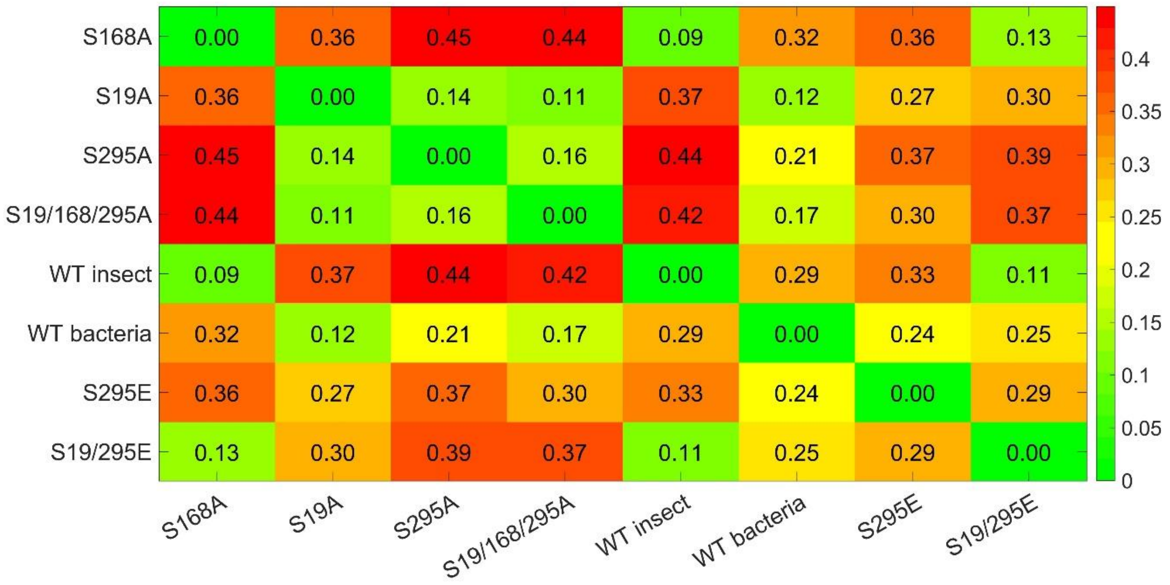

Figure S5. Heat maps of Kolmogorov-Smirnov (KS) statistic for the Utr N-R3 constructs. KS test metrics which compare the distribution of unfolding forces for different UtrN-R3 constructs. The color-bars (heat maps) represent the KS metric with a 0-value indicating similarity and value of 1 representing maximum dissimilarity.

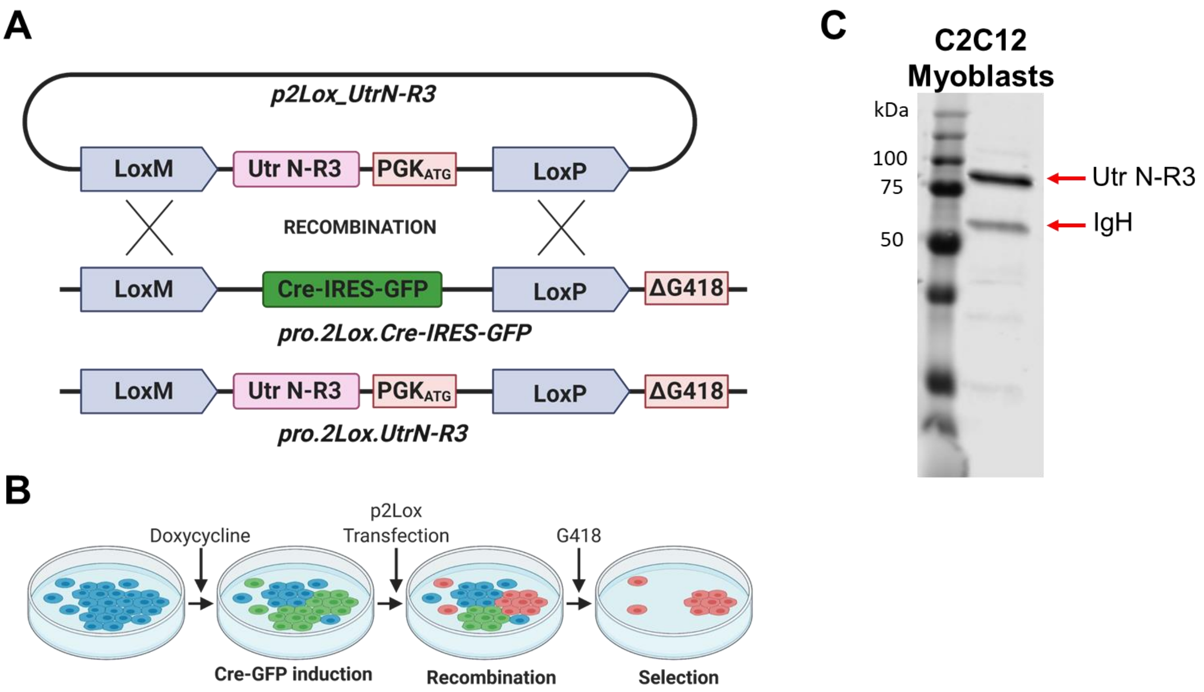

Figure S6. Generation of an inducible UtrN-R3 C2C12 cell line. A. Schematic representation of the p2Lox_UtrN-R3 plasmid and inducible cassette exchange locus before and after recombination. **B.** Sequential steps to generate inducible (i) isogenic C2C12 line that overexpresses Flag-UtrN-R3 protein, from induction to selection. **C.** Representative western blot of final purified UtrN-R3 product from C2C12 mammalian cells. Lysate probed for Flag-UtrN-R3 with FLAG antibody.

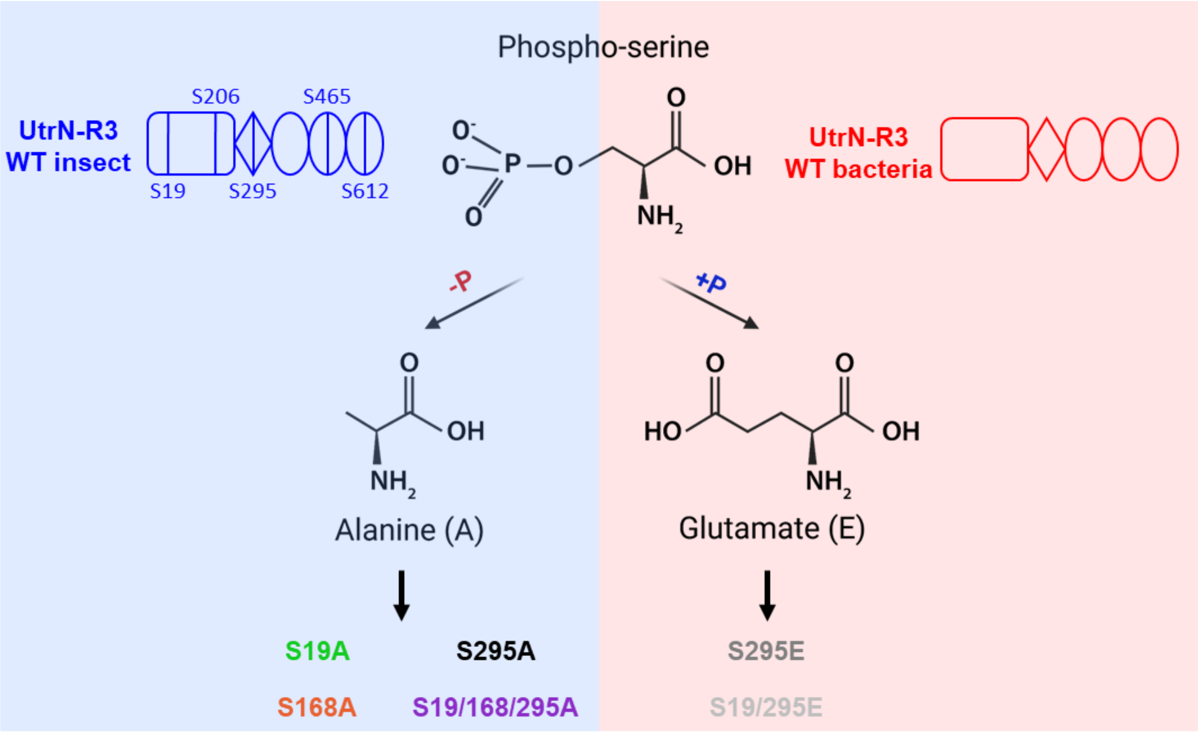

Figure S7. UtrN-R3 phospho-mutant library. Schematics of phospho-mutants that could potentially modulate unfolding forces by mutating serine residues to alanine which ablates phosphorylation (left), or glutamate which mimics phosphorylation (right).

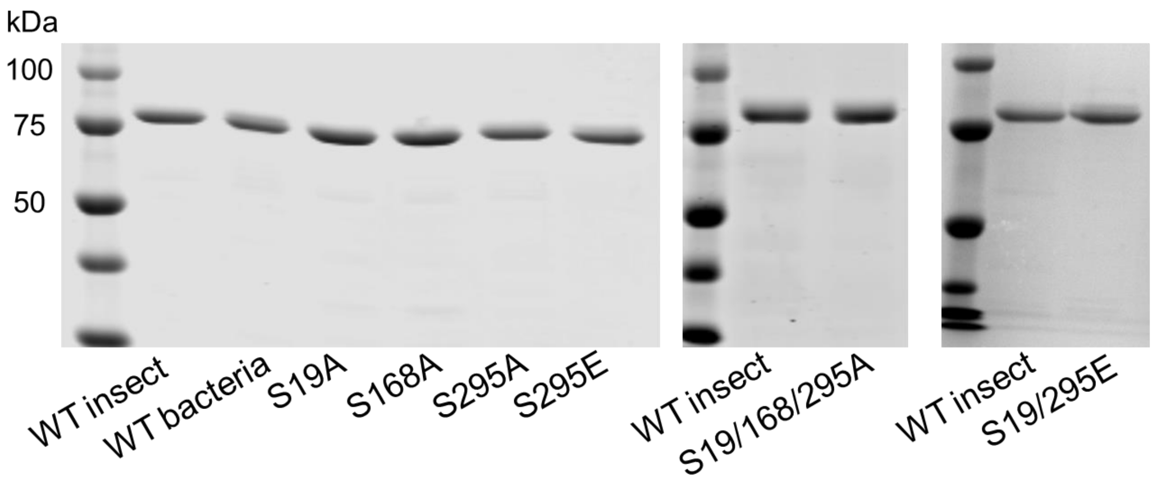

Figure S8. Purified UtrN-R3 constructs. Coomassie-stained gel of purified UtrN-R3 WT and mutant constructs expressed in insect (WT insect, S19A, S168A, 295A, S19/168/295A) and bacterial cells (WT bacteria and S295E).

```
        10         20         30         40         50         60
MDYKDDDDKA KYGDLEARPD DGQNEFSDII KSRSDEHNDV QKKTFTKWIN ARFSKSGKPP

        70         80         90        100        110        120
ISDMFSDLKD GRKLLDLLEG LTGTSLPKER GSTRVHALNN VNRVLQVLHQ NNVDLVNIGG

       130        140        150        160        170        180
TDIVDGNPKL TLGLLWSIIL HWQVKDVMKD IMSDLQQTNS EKILLSWVRQ TTRPYSQVNV

       190        200        210        220        230        240
LNFTTSWTDG LAFNAVLHRH KPDLFSWDRV VKMSPIERLE HAFSKAHTYL GIEKLLDPED

       250        260        270        280        290        300
VAVHLPDKKS IIMYLTSLFE VLPQQVTIDA IREVETLPRK YKKECEEEEI HIQSAVLAEE

       310        320        330        340        350        360
GQS PRAETPS TVTEVDMDLD SYQIALEEVL TWLLSAEDTF QEQDDISDDV EEVKEQFATH

       370        380        390        400        410        420
ETFMMELTAH QSSVGSVLQA GNQLMTQGTL SEEEEFEIQE QMTLLNARWE ALRVESMERQ

       430        440        450        460        470        480
SRLHDALMEL QKKQLQQLSS WLALTEERIQ KMESLPLGDD LPSLQKLLQE HKSLQNDLEA

       490        500        510        520        530        540
EQVKVNSLTH MVVIVDENSG ESATALLEDQ LQKLGERWTA VCRWTEERWN RLQEISILWQ

       550        560        570        580        590        600
ELLEEQCLLE AWLTEKEEAL NKVQTSNFKD QKELSVSVRR LAILKEDMEM KRQTLDQLSE

       610        620        630        640
IGQDVGQLLS NPKASKKMNS DSEELTQRWD SLVQRLEDSS NQVTQAQAM
```

Confidently assigned Phosphorylation

Not confidently assigned Phosphorylation

Predicted Phosphorylation

Figure S9. FLAG-UtrN-R3 Phosphorylation Site Predictions. Predictions obtained using NetPhos 3.1 and NetPhorest 2.1 online phosphorylation prediction software. Numbering of residues is off by 8 due to the presence of the FLAG tag (underscored sequence).

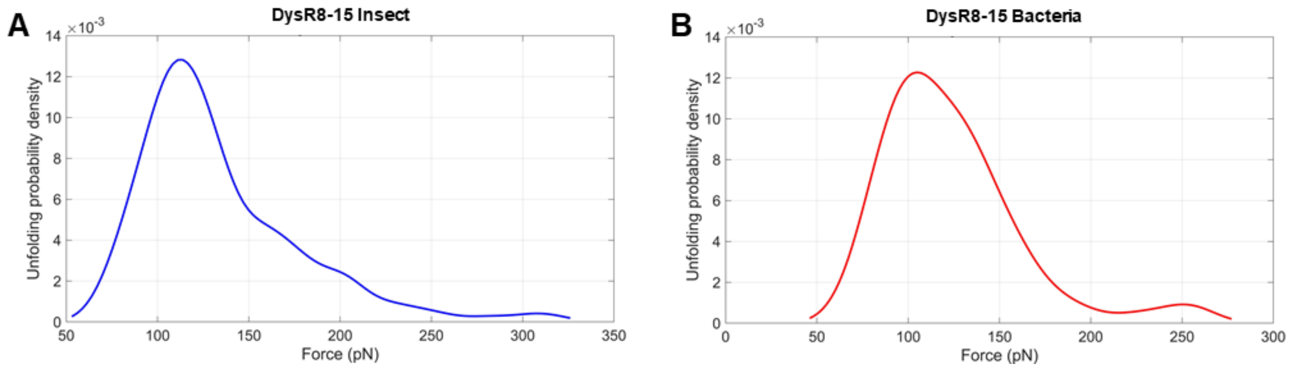

Figure S10. Unfolding force distributions for DysR8-15. Probability density distributions of unfolding forces vs unfolding force for purified **A.** insect and **B.** bacterial DysR8-15. Data collected from total unfolding events (>160 per construct), from N≥3 biological replicates.

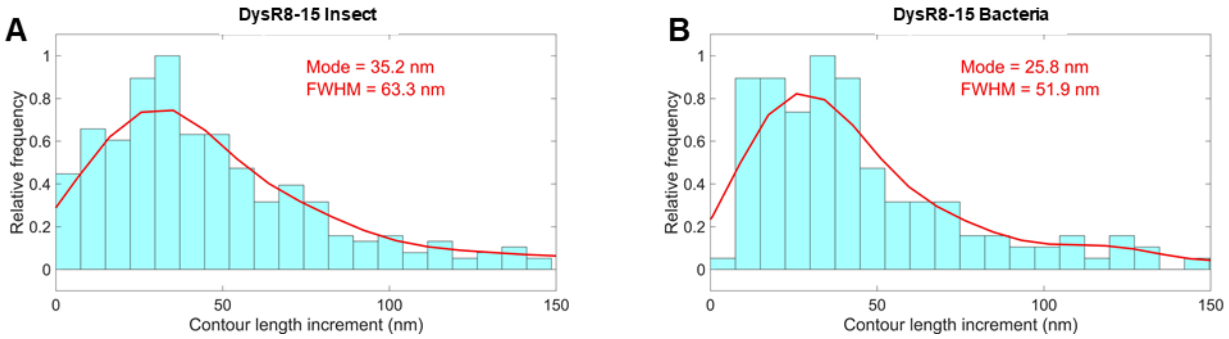

Figure S11. Contour length increment histograms for DysR8-15 constructs. The most probable contour length increments range between 25.8 to 35.2 nm.

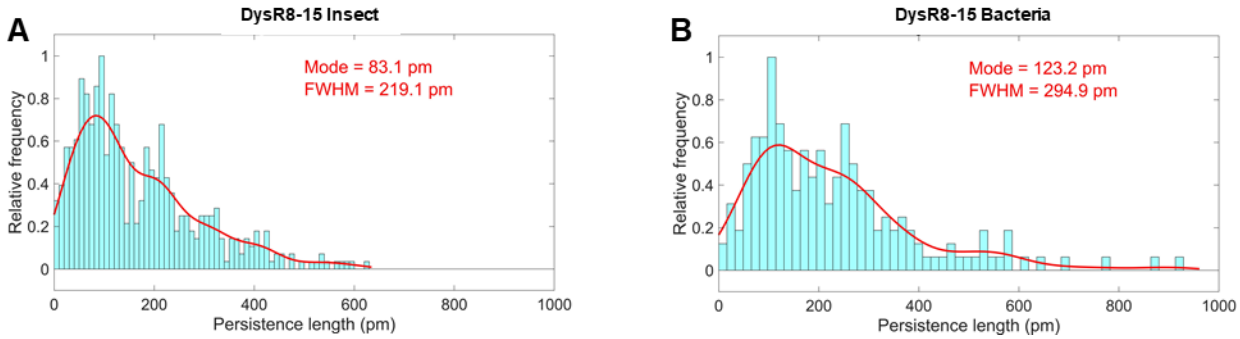

Figure S12. Persistence length increment histograms for DysR8-15 constructs. The most probable persistence lengths were found to be between 83.1 to 123.2 pm for the utrophin constructs.
